# Key to the Tribes and Genera of Deltocephaline Leafhoppers (Auchenorrhyncha, Hemiptera, Cicadellidae) of Pakistan

**DOI:** 10.3897/zookeys.104.906

**Published:** 2011-06-13

**Authors:** Imran Khatri, Maqsood Anwar Rustamani

**Affiliations:** Department of Entomology Sindh Agriculture University Tando Jam, Pakistan

**Keywords:** Deltocephalinae, taxonomy, morphology

## Abstract

A key with accompanying figures is provided for the 14 tribes and 35 genera of Deltocephalinae (Cicadellidae) from Pakistan.

## Introduction

Deltocephalinae is the largest and most diverse subfamily of Cicadellidae with 6200 described species placed in over 850 genera (McKamey, in press), in 36 tribes (Zahniser and Dietrich 2010). Members of the subfamily are also important vectors of plant diseases (Weintraub and Beanland 2006) and account for 117 of the 151 cicadellid vector species listed by Nielson (1968).

Early leafhopper taxonomic work in Pakistan (from 1960 onwards) focused on Typhlocybinae. Work on Deltocephalinae was scattered in various publications (Ahmed 1986, Ahmed and Aziz 1988, Ahmed and Rao 1986, Ahmed et al. 1988, Ara and Ahmed 1988, Fatima et al. 1995, 1997, 1998, Mahmood 1979, 1980, Mahmood and Aziz 1979, Mahmood and Meher 1973, Mahmood et al. 1972), but a thorough review of this literature was given by Khatri and Webb (2010). These authors also provided a checklist, new combinations, new species, new synonymy and new record and a key to the 14 tribes.

In the present paper we revise the above tribal key to include Mukariini, following its recent discovery in Pakistan (Khatri and Webb 2011) and its inclusion in the subfamily by Zahniser and Dietrich (2010), together with Drabescini (Paraboloponina) and Penthimiini, also included by Zahniser and Dietrich (2010). Drabescini is represented in Pakistan by *Dryadomorpha pallida* Kirkaldy (1906: 336), recorded from Pakistan as *Rhombopsis viridis* Pruthi see (Webb 1981) and Penthimiini, represented by *Neodartus acocephaloides* Melichar (1903: 163), from Hafizabad and *Penthimia compacta* Walker (1851: 842) from Murree Hills (National Pusa Collection, IARI, New Delhi, pers. com. Dr. C.A. Viraktamath).

An additional genus and species, omitted by Khatri and Webb (2010), is also included: *Gurawa minorcephala* Pruthi (1930: 29) (described from Pakistan: Murree Hills) and following Zahniser (2008) is placed in Chiasmini. We here follow Khatri and Webb’s (2010) broad concept of Athysanini to include *Osbornellus* Ball and *Scaphoideus* Uhler which were left unplaced to tribe by Zahniser and Dietrich (2010).

One other species, *Hengchunia pakistanica* Asche & Webb 1994 (from India) was erroneously recorded from Pakistan.

In total 35 known genera are included in the key. Figures are also provided and additional figures can be found in Khatri and Webb (2010).

## Key to Deltocephalinae tribes and genera from Pakistan

Note: The characters given in the key will separate the Pakistan genera but not necessarily the tribes on a wider distribution. As the genera in the following key are grouped by tribe and as some of these are based only on the male genitalia, several genera in the latter part of the key are based only on the male.

**Table:** 

1	Anterior margin of head with transverse striations or carinae (Figs 1j, l)	2
–	Anterior margin of head smooth or shagreen	7
2	Clypellus elongate, extending beyond margin of genae, tapered to apex and slightly bent subapically under head.	(Grypotini).....3
–	Clypellus short not extending beyond margin of genae	4
3	Vertex of head medially longer than next to eyes; ocelli equidistant between eye and median line of head; 5–6 mm in length	*Sohipona* Ghauri & Viraktamath
–	Vertex of head of uniform length; ocelli placed slightly closer to eye than to median line; less than 5 mm in length	*Pinopona* Viraktamath & Sohi
4	Antennae very long, extending beyond midlength of body, situated at upper corner of eyes (Fig. 1k)	Drabescini (Paraboloponina) *Dryadomorpha* Kirkaldy
–	Antennae short, not reaching midlength of body, situated below upper corner of eyes	5
5	Ocelli on foremargin of head bound both dorsally and ventrally by at least one carina (fig. 1j)	6
–	Ocelli located before foremargin of head, with carinae only ventrally	Penthimiini *Penthimia* Distant
6	Head depressed anteriorly (Fig. 1j); forewing venation reticulate; aedeagus with one shaft	Penthimiini *Neodartus* Melichar
–	Head not depressed anteriorly, forewing venation not reticulate; aedeagus with two shafts (Fig. 3b)	Mukariini (*Mukaria* Distant)
7	Robust species; vertex distinctly broader than long and only slightly longer medially than next to eyes (Fig. 1b); forewing appendix extending to outer apical cell (Fig. 1r); subgenital plates fused to each other and to valve (Fig. 2m); style apical process expanded apically; aedeagus fused to connective (Fig. 3e)	Goniagnathini (*Goniagnathus* Fieber)
–	Without the above combination of characters	8
8	Genae of face broad (Fig. 1h), visible in dorsal view (Fig. 1a).	Scaphytopiini.....9
–	Genae of face narrow, not visible dorsally	11
9	Forewing obliquely truncate at apex; green species with red longitudinal stripes on head, thorax and forewings (Fig. 1e)	*Varta* Distant
–	Forewing rounded at apex (Fig. 1o); colour not as above	10
10	Pronotum with lateral carina	*Grammacephalus* Haupt
–	Pronotum without lateral carina	*Masiripius* Dlabola
11	Face with laterofrontal sutures directed mediad of and terminating distad of corresponding ocelli (Fig. 1g). Head, particularly in female, somewhat spatulate. Ovipositor with second valvulae, lacking teeth (Fig. 3l).	Hecalini.....12
–	Without the above combination of characters	13
12	Green species, male pygofer without caudal marginal stout setae (Fig. 2d)	*Hecalus* Stål
–	Brown species; male pygofer with caudal marginal stout setae	*Glossocratus* Fieber
13	Vertex narrow basally (Fig. 1f). Male pygofer elongate, without a membranous laterobasal slit, with a lateroposterior triangular process (Fig. 2f); valve long; subgenital plate short; connective with stem two pronged apically, arms parallel (Fig. 2o); aedeagal shaft whip-like (Fig. 3d). Ovipositor with second valvulae lacking teeth (Fig. 3m)	Stenometopiini (*Stirellus* Osborn & Ball)
–	Without the above combination of characters	14
14	Forewing brachypterous or if macropterous then appendix (when present) extending to fourth (outer) apical cell (Fig. 1m). Aedeagal shaft hinged (Fig. 3a) or not hinged; connective with arms looped (Fig. 2n). Ovipositor with sculpture granular, not extending to dorsal margin (Fig. 3n).	Chiasmini.....15
–	Forewing appendix extending to second apical cell (Fig. 1n). Aedeagal shaft not hinged; connective with arms looped or divergent. Ovipositor with sculpture reticulate, extending to dorsal margin	19
15	Forewing with two subapical cells (Figs 1m)	*Aconurella* Ribaut
–	Forewing with three subapical cells (Figs ln, o)	16
16	Head margin depressed in lateral view	17
–	Head margin rounded in lateral view	18
17	Forewing lacking appendix. Aedeagal shaft not hinged at base with atrium; compressed in apical region with serrated margin	*Gurawa* Distant
–	Forewing when well developed, with appendix. Aedeagal shaft hinged at base with atrium (Fig. 3a), shaft cylindrical and lacking serration	*Chiasmus* Mulsant & Rey
18	Predominantly green species	*Nephotettix* Matsumura
–	Predominantly pale brown species	*Exitianus* Ball
19	Forewing with two subapical cells (Fig. 1q). Connective with arms divergent.	Macrostelini.....20
–	Forewing with three subapical cells (Fig. 1n, o), sometimes outer subapical cell subdivided (Fig. 1s), or if two subapical cells connective with arms convergent apically	22
20	Vertex of head short, of uniform length, more than 4 times wider than long (Fig. 1c)	*Balclutha* Kirkaldy
–	Vertex longer medially than next to eyes, twice or less as wide as medial length	21
21	Head and thorax golden yellow, vertex with two round dark brown spots (Fig. 1d). Male pygofer with long, slender hook-like process; without marginal comb-like serrations (Fig. 2a)	*Cicadulina* China
–	Head and thorax not as above. Male pygofer without above process; with comb-like serrations on caudal margin (Fig. 2b)	*Macrosteles* Fieber
22	Aedeagus with two shafts.	Opsiini.....23
–	Aedeagus with one shaft	25
23	Aedeagal shafts fused in basal half, then divergent describing a circle (Figs 3c)	*Neoaliturus* (*Circulifer*) Distant
–	Aedeagal shafts separate at base (Figs 3g, 3h)	24
24	Aedeagal shaft with ventral pair of processes (Figs 3g, 3j)	*Opsius* Fieber
–	Aedeagal shaft without ventral pair of processes (Fig. 3h)	*Orosius* Distant
25	Connective with arms divergent (Fig. 2k).	Athysanini.....26
–	Connective with arms at most only divergent basally, convergent apically	32
26	Vertex acutely pointed, disc depressed; forewing venation reticulate (Fig. 1s)	*Platymetopius* Burmeister
–	Vertex and forewings not as above	27
27	Three to four cross veins from outer apical cell reaching costal margin (Fig. 1n)	28
–	At most two cross veins from outer apical cell reaching costal margin	29
28	Male connective with paraphyses (Fig. 2g)	*Scaphoideus* Uhler
–	Male connective without paraphyses (Fig. 2k)	*Bampurius* Dlabola
29	Male subgenital plates with mesal sclerotized process (Fig. 2h)	*Neolimnus* Linnavuori
–	Male subgenital plates without mesal sclerotized process	30
30	Aedeagal shaft without basal processes, apophysis of style long, subequal to length of aedeagal shaft (Fig. 2e)	*Pseudosubhimalus* Ghauri
–	Aedeagal shaft with basal pair of dorsal (Fig. 3i) or ventral (Fig. 3k) processes, apophysis of style shorter than aedeagal shaft	31
31	Aedeagal shaft with basal processes arising on dorsal surface of shaft (Fig. 3i)	*Monobazus* Distant
–	Aedeagal shaft with basal process arising on ventro-lateral surface of shaft (Fig. 3k)	*Osbornellus* Ball
32	Connective fused to aedeagus (Fig. 3f).	Deltocephalini.....33
–	Connective not fused to aedeagus (Fig. 2i).	Paralimnini.....34
33	Vertex with transverse black stripe; pygofer with dorsal marginal appendage	*Paramesodes* Ishihara
–	Vertex without transverse black stripe; pygofer without appendage (Fig. 2c)	*Maiestas* Distant
34	Male subgenital plates short (Fig. 2i); mesal arm of style longer than outer arm (Fig. 2j)	*Psammotettix* Haupt
–	Male subgenital plate longer; mesal arm of style shorter than outer arm (Fig. 2l)	*Jilinga* Ghauri

**Figure 1.**
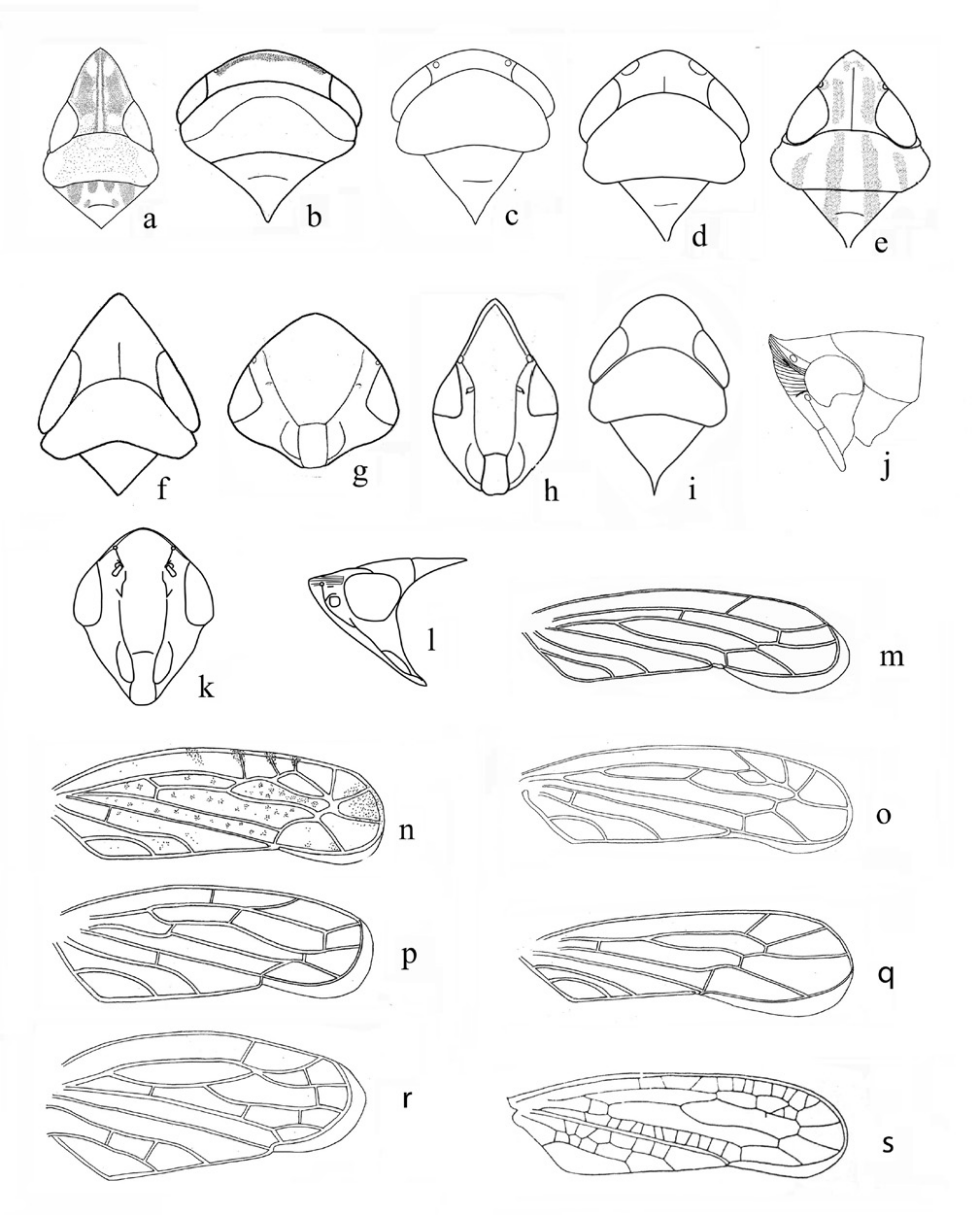
**a–f, i** (head and thorax dorsal view); **g, h, k** (face); **j, l** (head and thorax lateral view); **m–s** (right forewing) **A** *Grammacephalus indicus* Viraktamath & Murthy **B** *Goniagnathus (T.) quadripinnatus* Dash & Viraktamath **C** B*alclutha incisa* (Matsumura, 1902) **D** *Cicadulina bipunctata* Ghauri **E** *Varta rubrofasciata* Distant **F** *Stirellus lahorensis* (Distant) **G** *Hecalus sindhensis* (Ahmed & Aziz) **H** *Grammacephalus indicus* Viraktamath & Murthy **I** *Mukaria splendida* Distant **J** *Neodartus acocephaloides* Melichar **K, L** *Dryadomorpha pallida* Kirkaldy **M** *Aconurella prolixa* (Lethierry) **N** *Bampurius pakistanicus* Khatri & Webb **O** *Grammacephalus indicus* Viraktamath & Murthy **P** *Chiasmus* sp. **Q** *Macrosteles indrina* (Pruthi) **R** *Goniagnathus (T.) quadripinnatus* Dash & Viraktamath **S** *Platymetopius* sp.

**Figure 2.**
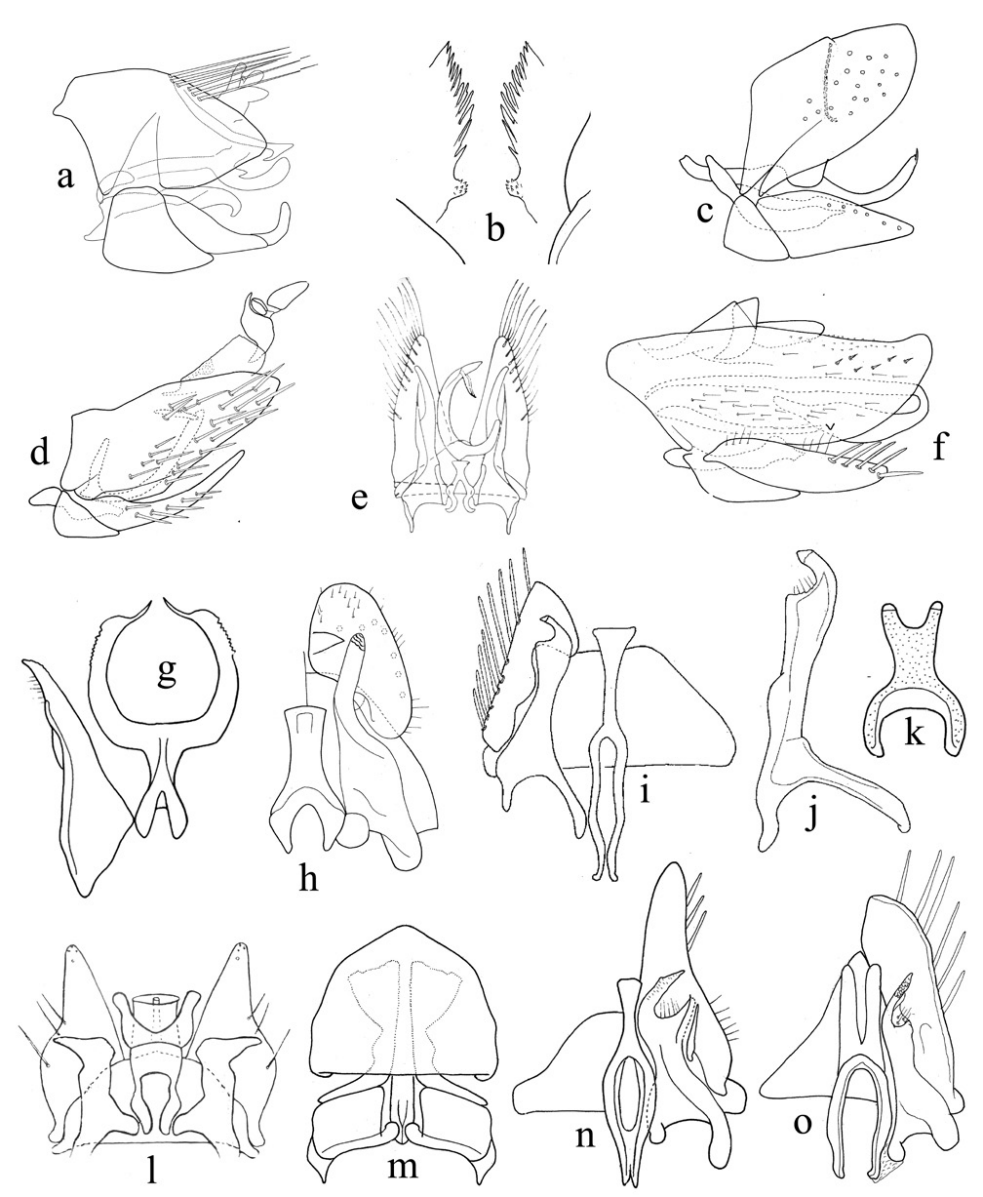
**a–o** male genitalia. **a, c, d, f** (genital capsule); **b** (pygofer, caudal view); **e** (genital capsule, internal view); **g** (connective and style); **h, i, n, o** (valve, style and connective, dorsal view). **j** (style); **k** (connective); **l** (valve, styles, subgenital plates, connective and base of aedeagus); **m** (fused subgenital plates and valve (setae omitted), styles and base of connective). **A** *Cicadulina bipunctata* Ghauri **B** *Macrosteles indrina* (Pruthi) **C** *Maiestas pruthii* (Metcalf) **D** *Hecalus sindhensis* (Ahmed & Aziz) **E** *Pseudosubhimalus bicolor* Pruthi **F** *Stirellus lahorensis* (Distant) **G** *Scaphoideus harlani* Kitbamroong & Freytag **H** *Neolimnus quadricornis* Khatri & Webb **I, J** *Psammotettix emarginata* Singh **K** *Bampurius pakistanicus* Khatri & Webb **L** *Jilinga gopii* (Pruthi) **M** *Goniagnathus (T.) quadripinnatus* Dash & Viraktamath **N** *Aconurella prolixa* (Lethierry) **O** *Stirellus lahorensis* (Distant).

**Figure 3.**
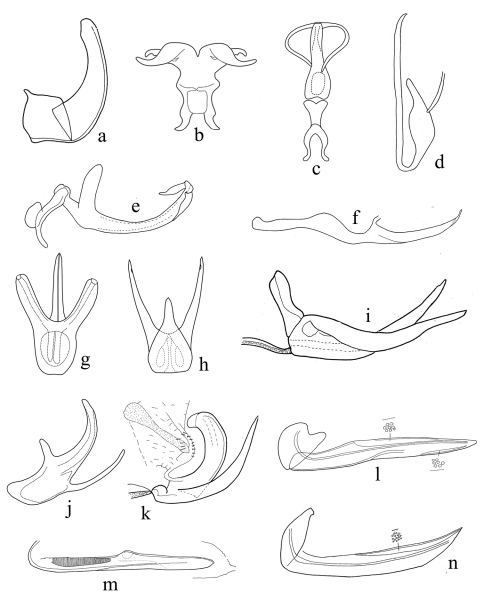
**a–n** male and female genitalia; **a, d, g–j** (aedeagus); **b–c, e–f** (aedeagus and connective); **k** aedeagus, apex of connective and pygofer; **l, n** (first valvulae); **m** (second valvulae). **A** *Aconurella prolixa* (Lethierry) **B** *Mukaria splendida* Distant **C** *Neoaliturus (Circulifer) tenellus* (Baker) **D** *Stirellus lahorensis* (Distant, 1918) **E** *Goniagnathus (T.) quadripinnatus* Dash & Viraktamath, 2001 **F** *Maiestas tareni* (Dash & Viraktamath) **G** *Opsius versicolor* (Distant) **H** *Orosius albicinctus* Distant **I** *Monobazus dissimilis* (Distant) **J** *Opsius versicolor* (Distant) **K** *Osbornellus (Mavromoustaca) macchiae* (Lindberg) **L** *Hecalus sindhensis* (Ahmad & Aziz) **M** *Stirellus lahorensis* (Distant) **N** *Aconurella prolixa* (Lethierry).
